# Realizing Efficient Security and Privacy in IoT Networks

**DOI:** 10.3390/s20092609

**Published:** 2020-05-03

**Authors:** Joseph Henry Anajemba, Yue Tang, Celestine Iwendi, Akpesiri Ohwoekevwo, Gautam Srivastava, Ohyun Jo

**Affiliations:** 1 Department of Communication Engineering, College of Internet of Things, Hohai University, Nanjing 210098, China; herinopallazo@ieee.org (J.H.A.); 20141933@hhu.edu.cn (Y.T.); 2 Department of Electronics BCC of Central South University of Forestry and Technology, Changsha 410004, China; celestine.iwendi@ieee.org; 3 Computer Science and Technology, Xidian University, Xi’an 710126, China; gamaliel@stu.xidian.edu.cn; 4 Research Centre of Interneural Computing, China Medical University, Taichung 404472, Taiwan; 5 College of Information and Electrical Engineering, Asia University, Taichung 41354, Taiwan; 6 Department of Computer Science, College of Electrical and Computer Engineering, Chungbuk National University, Cheongju-si 28644, Korea

**Keywords:** privacy capacity, IoT, 5G, physical layer security, MIMOME, jamming

## Abstract

In recent times, security and privacy at the physical (PHY) layer has been a major issue of several communication technologies which comprise the internet of things (IoT) and mostly, the emerging fifth-generation (5G) cellular network. The most real-world PHY security challenge stems from the fact that the passive eavesdropper’s information is unavailable to the genuine source and destination (transmitter/receiver) nodes in the network. Without this information, it is difficult to optimize the broadcasting parameters. Therefore, in this research, we propose an efficient sequential convex estimation optimization (SCEO) algorithm to mitigate this challenge and improve the security of physical layer (PHY) in a three-node wireless communication network. The results of our experiments indicate that by using the SCEO algorithm, an optimal performance and enhanced convergence is achieved in the transmission. However, considering possible security challenges envisaged when a multiple eavesdropper is active in a network, we expanded our research to develop a swift privacy rate optimization algorithm for a multiple-input, multiple-output, multiple-eavesdropper (MIMOME) scenario as it is applicable to security in IoT and 5G technologies. The result of the investigation show that the algorithm executes significantly with minimal complexity when compared with nonoptimal parameters. We further employed the use of rate constraint together with self-interference of the full-duplex transmission at the receiving node, which makes the performance of our technique outstanding when compared with previous studies.

## 1. Introduction

With the recent swift advancement of wireless communication networks and the advent of the fifth generation (5G) cellular network, interconnected devices are embedded into the environment through the IoT paradigm to enhance constant quality of service (QoS) and connectivity [1]. However, security of wireless transmissions has become a vital concern [2]. Unfortunately, in wireless technology, security risks are unavoidably inherent. Recently, network intrusion and eavesdropping, known as Eves, has become the major cradle of security risks in 5G wireless communications. The resources (e.g., battery) of most IoT devices (such as handheld/mobile communicating devices) are constrained, thus resulting in the devices having limited power for transmission, which may warrant the use of frailer cryptographic techniques as power savers. Therefore, devices may be prone to several attacks by some prevailing adversaries.

Security in wireless networks is typically employed through cryptographic methods using the upper layers of the open system interconnection (OSI) model [2,3]. However, these adversaries, which are considered as unauthorized users or network Eves, may infiltrate the network system, exhaust the networks bandwidth, taint the transmission data, reduce transmission performance, and inject data harms that thwart easy access of network data by authentic users. As a result of wireless link’s unprotected nature, most wireless connectivity is susceptible which makes it easy for them to be attacked by jamming technology. These jamming attacks can result in a problem of Denial-of-Service (DoS), which can cause numerous other higher-layer security glitches in IoT and 5G technologies [3].

The 5G wireless network is predicted to activate the smart hyperlinked environment, enhancing the evolution and the growth of several sectors, such as energy and railway, where huge amounts of accessibility and reliability is essential. In contrast, this evolution may warrant that future mobile transmitting devices be exposed to cyber-attacks, which can destabilize system accessibility [4]. Because of the nature of wireless communications, vulnerability to Eve’s attacks is inevitable. Therefore, transmission confidentiality cannot be guaranteed. In this paper, our work is focused on utilizing the networks physical layer to address countermeasures towards tackling confidentiality attacks in wireless network.

5G communications will headline what is being called the 4th Industrial Revolution, where the mobile wireless broadband, pervasive sensing, and artificial intelligence (AI) promises to lead to major changes in academia, industry, and society itself. This coming generation of wireless communications globally focuses on many aspects as standards, policy, and infrastructure are still being shaped. 5G definitely promises to make the IoT network a reality.

In wireless networks, confidentiality attacks mostly stem from jamming. This is considered as the interruption of the flow in wireless transmissions by diminishing the signal-to-noise ratio (SNR) at the receiver end over a wireless signal’s transmission interference. This clearly varies from normal network interferences because it explains a deliberate injection of wireless signals into an existing transmission with the intention of interrupting communications. However, network interference is said to be an accidental kind of disruption during transmission [5]. Recently, through conventional means, the implementation of different encryption approaches has been used to handle this security challenge at the advanced communications layers. That being said, a lot of attention has been drawn towards the security of the physical (PHY) layer lately. From a PHY security perspective, the authors of [6] investigated the impact of saturation nonlinear energy harvesting (EH) and activation threshold on the multiuser wireless powered sensor networks (WPSNs) from the physical layer security (PLS) perspective, and with respect to the generalized multiuser scheduling (GMS), they examined the improvement in the secrecy performance in WPNs. Their work explored and presented an exact closed-form expressions for secrecy outage probability (SOP) under linear EH (LEH), saturation nonlinear EH (SNEH), and saturation nonlinear EH with activation threshold (SNAT), respectively, through finding a solution for the maximization problem of secure energy efficiency (SEE).

The basis for tackling jamming attacks is hinged on Wyner’s architecture. This architecture presented and demonstrated a wiretap channel when the channel of the eavesdropper (Eve) is a tainted version of the authentic receiver’s channel, a secret message can be sent from the transmitter to the destination, while Eve is kept unaware of the content of the transmitted message [7]. The concept of privacy capacity is described as the optimal attainable rate of transmission of private data from the transmitting source (node) to its receiving destination. In [8], the Wyner’s wiretap method was generalized by assuming the private message transmission over channels broadcast. Lately, substantial studies have analyzed privacy in wiretap channels under multiple antennas networks [9,10,11]. Particularly, the authors of [11,12] described the performance capacity of privacy in a multiple-input, multiple-output (MIMO) wiretap transmission channel. However, the authors of [13] investigated a joint effect of multiple jamming signals and noise at the eavesdropper in a MIMO network by assuming the implementation of antenna selection technique by the transmitter, while both the eavesdropper and legitimate receiver make use of a maximal-ratio combining scheme to achieve spatial diversity in reception. Their results show the effects of imperfect feedback and other key system parameters on the secrecy performance.

Basically, security at the PHY layer guarantees an optimal level of transmission privacy against Eve’s as well as establishing an anticipated reception standard and quality as expected by the receiver(s). This research area comprises both signal processing analysis and theoretical information study. Although the latter involves more bounds and asymptotical limits, the former inclines towards innovating primal designs of algorithms and architectures to tackle security issues in a wireless network. Therefore, the focus and motivations of this paper is wrapped around the former with respect to Eve’s growing quantity of transmitting antennas on privacy.

All the above-mentioned studies, except for [10,11], failed to explore a MIMOME scenario wish illustrates the effects of Eve’s growing quantity of transmitting antennas on privacy. Although the authors of [6,11,13] proposed proficient privacy, their technique is reliant only on the assumption of a scenario where several factors such as the quantity of Eve’s antennas that are accessible for transmitter usage for artificial noise subspace, are constrained in a MIMO and MIMOME network. By implication, it is essential that Eve’s multiple antenna usage needs be considered and addressed. In this research, this is believed to be an issue of importance, therefore these problems are first extensively described, then techniques to tackle such problems are investigated and proposed.The main contributions of this research are as follows.

- A primal outlook into the investigation and optimization of wireless communications security with respect to IoT networks is presented.
- Using a mathematical model, an analysis of a novel transmission system where the eavesdroppers attack is tackled by injecting artificial noise which is transmitted by the receiver who has full-duplex aptitude with the same frequency in the channel, which reduces Eve’s reception quality.
- Proposal of a new jamming mitigation technique and developed a sequential convex estimation and optimization (SCEO) algorithm for an optimized and enhanced privacy to solve the optimization problem in a three-node network where network users do not have knowledge of Eve’s channel state information (CSI).
- The vulnerability of the proposed privacy enhancing scheme to Eve’s increasing number of antennas is characterized and explored, while the performance of the proposed algorithm in a three-node network is established.
- Finally, because privacy capacity in a three-node network have been over-studied by several authors, this research is expanded to cover a MIMOME scenario, justifying its applicability in secured IoT network transmission.

The remainder of this paper is organized as follows. The privacy capacity model in wireless networks is described in Section 2. Review of recent works related to the research is presented in Section 3. System model is described in Section 4. Numerical analysis and results are provided in Section 5, and finally conclusions are outlined in Section 6.

## 2. Privacy Capacity Model in Wireless Networks

**Figure 1:**
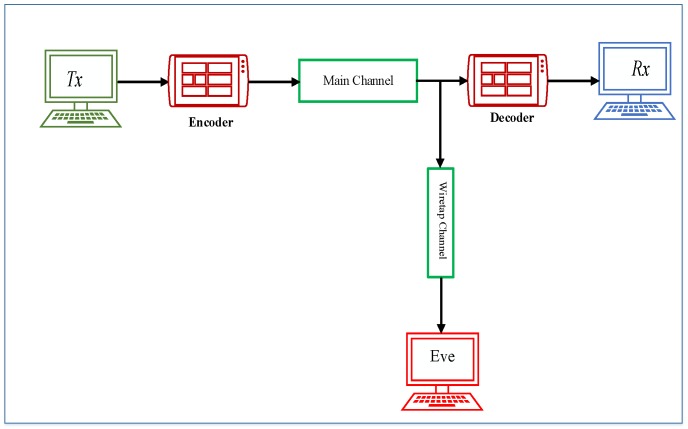
A Wiretap Channel for Privacy Capacity model.

In this section, the privacy capacity model of the transmitting network is expressed. In the privacy capacity model of the network, it is assumed that the malicious node may eavesdrop the source as well as well as the receiver. However, in order to get a full use of the signals transmitted from the transmitter, the eavesdropper must be fully synchronized in the network. According to traditional privacy definitions in [8], the channel of communication can be modeled as a channel of broadcast in line with the wiretap channel as illustrated in Figure 1.

Considering the wiretap channel, the transmitting channel’s message is represented as $a^{n}\in A^{n}$ and is encoded and broadcasted as a codeword $c^{m}\in C^{m}$. The receiver $\left(R_{x}\right)$ and eavesdropper (Eve) receive $b^{m}\in B^{m}$ and $e^{m}\in E^{m}$, respectively. Eve’s received information via her receiving signal is then modeled and described as in Equation (1),

(1) $$I\left(e^{m};a^{n}\right)=g\left(a^{n}\right)-g\left(a^{n}\left|e^{m}\right.\right)$$

where $I(e^{m};a^{n})$ represents the mutual information shared by the transmitter and the legitimate receivers, while *g* is considered as the entropy. As long as Eve cannot decode any bit of the transmitted information, then perfect privacy is achieved. Thus, Equation (2),

(2) $$I\left(e^{m};a^{n}\right)=0↔g\left(a^{n}\right)=g\left(a^{n}\left|e^{m}\right.\right)$$

This implies that the quantity of uncertainty about Eve’s private information is not altered after $e^{m}$ is received.

By definition, the probability of experiencing an error $P_{i}$ in the message estimation of message $a^{n}$, and ${\hat{a}}^{n}$ is defined as the estimate of $a^{n}$; therefore,

(3) $$P_{i}=P\left\{a^{n}\neq {\hat{a}}^{n}\right\}.$$

Eve’s rate of uncertainty about message $a^{n}$ is term as the rate of equivocation and can be described as

(4) $$Q_{i}=\frac{1}{m}g\left(a^{n}\left|e^{m}\right.\right)$$

where

(5) $$0\leq Q_{i}\leq g\left(a^{n}\right)/m$$

Evidently, if $Q_{i}=g\left(a^{n}\right)/m$, then perfect privacy, which is related to perfect privacy rate $Q_{s}$, is realized. For each ε>0, a particular $Q_{s}$ is assumed to be realizable, and there is a sequence of $\left(2^{mQ_{s}},m\right)$ codes such that for any m>m(ε) the following states are obtained.

(6) $$\left\{\begin{array}{c} P_{i}<\varepsilon \\ Q_{s}-\varepsilon <Q_{i} \end{array}\right.$$

The first state is the constraint for the realizable rate, whereas the second is the equivocation rate constraint which guarantees prefect privacy. In summary, privacy capacity $S_{c}$ is the optimal realizable rate of privacy in a network transmission. Thus, in [8], it is established that the difference between the main channel capacity $C_{mc}$ and the wiretap channel capacity $C_{wc}$ is the privacy capacity $S_{c}$, in other words,

(7) $$S_{c}={\left(C_{mc}-C_{wc}\right)}^{+},$$

as ${\left(.\right)}^{+}\overset{\Delta }{=}\max\left(0,.\right)$, where negative rate is meaningless. Although Wyner demonstrated this for the distinct no-memory channel, the principle that the capacity of privacy is the difference of the capacity of the legitimate channel and that of the eavesdropper’s is established to be accurate for several systems like in multiple-input, multiple-output (MIMO) set-ups [12,13].

### Notations

We denoted the column vectors and matrices by boldface letters (both at upper and lower cases), while the determinant, inverse of a matrix and column-wise vectorization of the matrix X are all represented as $X^{H},X^{-1},\left|X\right|$, respectively. In a diagonal vectorization, Y is denoted as the column vector of y, whereas the random variable of y is represented as $C_{y}\left[.\right]$ and the probability of event occurrence is denoted as P{X}. Using the same probability space, we defined the random variables Y and $Y_{m}$, if $Y_{m}$ converges to Y, we transposed $Y_{m}\overset{a.s}{\to }Y$ as m→∞. We used *I* to represent the matrix identity of supplied size, as I(y;z) represents the information which is mutually and randomly transmitted between y and x variables.

## 3. Related Works

In a wireless network, jammers intentionally introduce radio frequency (RF) interference to distort wireless communications. This is achieved by occupying the transmitting channel and keeping it busy, thereby triggering the transmitter to withdraw each time it senses a busy wireless channel, or a tainted signal acknowledged at the receiver’s end. In this section, in an attempt to understand the jamming attack on wireless networks, different kinds of jamming in wireless communications as proposed by several researchers are explored. Primarily, network jamming can occur in different ways based on the type of jammer. Therefore, different kinds of jammers and their jamming mechanisms are reviewed in this section.

The established secrecy capacity rate principle by Wyner in [8] preceded several other theoretical proposals by different researchers. So far, the wireless channels rate of secrecy has been explored from several perspectives like in [9,10]. The different perspectives explored are as follows, the fading channels secrecy [9,10,11], the analysis of Gaussian wiretap channels secrecy of [11], the multiple antenna systems secrecy [13,14,15], the broadcast channels secrecy [16], the analysis of secure degrees of freedom [17,18,19,20,21], and the secrecy of cooperative jamming techniques coupled with helpers or relays [22,23,24,25,26,27,28,29].

Additionally, the theoretical information [30] forms guarantees defined by secrecy capacity of the communication channel; the nodes that legitimate can also assume active approaches to improve their communication secrecy. One important approach for improving the secrecy of wireless communication was developed in [31]. The approach proposes that the transmitter (Alice) injects artificial noise into legitimate channels null-space together with the data signal. This scheme is intended to actively reduce the reception quality of any Eve’s presence without altering the quality of legitimate receivers (Bobs) channel. Several other works [32,33] have also investigated this approach. Equipping Bob with the capacities of full-duplex radio aptitude, which enables him broadcast jamming noise counter to Eve, while both Bob and Eve attempt to acquire information from Alice [34,35] is another approach. The combination of the aforementioned two jamming approaches is investigated in most part of this study to attain secrecy at higher levels. Moreover, this combination was examined in [36], as it proposed a cooperative optimization algorithm to generate decent parameters for the transmission. Separately, two different sets of antennas are considered for broadcasting and for full-duplex reception. Meanwhile, the research did not fully consider the residual self-interference at Bob. Further, several other works were interested with comparable systems in [37,38]. The system proposed in [39] presents a scenario with Alice and Bob communicating simultaneously and are furnished with full-duplex radio with full consideration of residual self-interference. The study target was to discover the maximum beamforming transmit trajectories for artificial noise and signal considering some constraints of quality of service (QoS) with Eve’s CSI recognizable to users. With Eve’s exact CSI unrecognizable to the user in [40], a closed-form lower bound on the ergodic rate of secrecy is realized. The authors of [41,42] considered a case of a single-antenna Bob and multiple antenna base station coupled with a colluding and non-colluding single-antenna Eves. As an alternative to beamforming, an antenna selection scheme is used by the study and it is of the research assumption that Eves are dispersed in accordance to the process of Poisson point.

For cooperative relaying networks, several authors have also investigated different techniques for the security of systems physical layers [43]. In cooperative relaying networks, even destinations or relays are used as assistants to offer jamming signals to complicate the eavesdropper’s transmission. This method is known as cooperative jamming. The noise-forwarding approach which was introduced in [24], and applied in a channel comprising of four-terminal relay-eavesdropper also considered a full-duplex relay, which independently transmits secret messages codewords which are targeted at complicating the eavesdropper’s transmission. The authors of [44] investigated a two-stage cooperative jamming scheme (TSCS) which involves multiple relay nodes acting as the single-antenna’s source node extension. The relays in this study do not transmit the information signals as they only function as a helper. However, for a single-antenna relay network, the authors of [45] proposed three different cooperative communication methods. On the other hand, for the second hop, they attempted to optimize secrecy by deriving a power allocation approach and its corresponding relay weights. The study in [46] investigated a decode-and-forward (DF) relays performance based on an optimal beamforming strategy; however, the investigation is limited because it only considered a transmission where the Eve monitors just a single connection linking the transmission destination and relay. The investigation of [47] was based on different privacy enhancing technologies (PETs) for IoT devices which have resulted in a much efficiency and convenience to our daily life. Their survey claim to have identified current state of improvement of the PETs in several turfs and also analyzed how the current technologies adhere with the modern legal ideologies and privacy criteria in curtailing the threats to privacy. A secured IoT-based healthcare system, which operates through the body sensor networks (BSN) architecture, was examined by the authors of [48]. The main focus of their system is to concurrently realize system robustness of transmission and efficiency within publicly transmitting IoT-based communication networks. Utilizing a vigorous utilize crypto-primitives, they constructed two communication schemes to ensure confidentiality in transmission and support entity authentication among smart objects. As most of the IoT data is relevant to personal privacy, it is necessary to pay attention to data transmission security. The authors of [49] investigated an IoT-oriented offloading method (IOM) which is enclave with privacy preservation to solve the problem of privacy in Cloudlet-enabled Wireless Metropolitan Area Networks (CWMAN). Their research and that of [50] adopted the non-dominated sorting differential evolution algorithm (NSDE) in order to optimize the multi-objective problem.

By contrast to the works analyzed above, the work in [51] proposed a cooperative jamming approach for a half-duplex two-hop wireless MIMO relay scheme where the eavesdropper can bug the channels throughout the phases of transmission. The study investigation considered both single and multiple streams of data transmissions. However, for jamming support, due to the absence of an “outer” helpers, the relay, destination, and source must depend on themselves. Whether the eavesdropper is in proximity to the source or the destination, the strategy guarantees that it is jammed. In this strategy, both the source and the destination nodes perform as provisional assistants for transmitting jamming signals throughout the phases of transmission where they are generally inactive.

In summary, the event of optimizing privacy without the users recognizing Eve’s CSI is sparsely contained in literature; however, a few works like [52,53,54,55,56,57] gave more attention to the use of only enough energy to authenticate a particular QoS for Bob and is this energy is estimated in terms of signal-to-interference-plus-noise-ratio (SINR). Using the remaining energy, artificial interference is generated to jam Eve not minding the effect of Eve’s location. This process is implemented in place of making attempts to optimize the rate of secrecy, which is impossible without the users having a knowledge of Eve’s CSI [58,59]. Furthermore, as new telecommunication technologies emerge, the use of several security and privacy techniques proposed earlier becomes over exploited and obsolete; therefore, it is vital to develop advanced state-of-the-art techniques and algorithms that can mitigate against network jamming and eavesdropping attacks and ensure constant quality of service (QoS) in the network. Considering that this is the optimal focus of this research, therefore, the relevance of this research to cutting-edge telecommunication technologies like the IoT and 5G cellular network cannot be underestimated.

## 4. System Model

**Figure 2:**
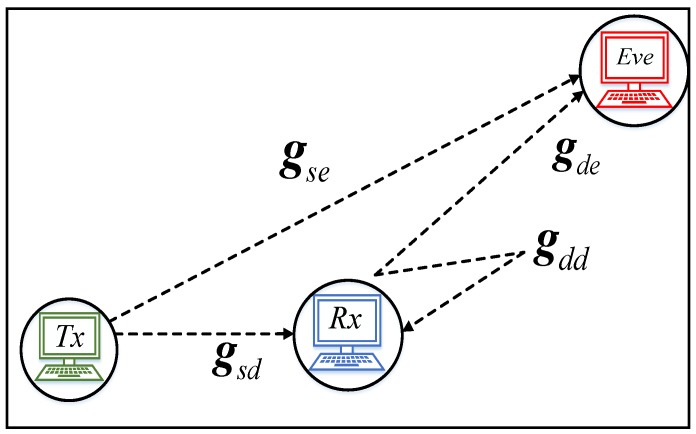
A single-antenna three-node wireless transmission with a full-duplex destination.

In this section, the system model is described. There are similarities in the model to what was given in [8]. For the set-up, a three-node-based wireless transmission is illustrated in Figure 2.

In the set-up, all three nodes are attached to a single transmitting antenna. The intention of the transmitter (Source) is to broadcast some private information to the receiver (Destination) while Eve (a passive eavesdropper) attempts to gain access to the sensitive private information. We assume that each link channel entails several *M* orthogonal subcarriers and each subcarrier fading is flat. Tackling eavesdropping attacks in this set-up entails that artificial noise is broadcasted by $R_{x}$ with the same frequency and in the same channel, which reduces Eve’s reception quality. Considering that this jamming attack operates at the same frequency and time in which information is transmitted from $T_{x}$ to $R_{x}$; thus, it is assumed that $R_{x}$ has full-duplex aptitude. It is well known that there is no perfect full-duplex system, therefore $R_{x}$ always manifests some level of residual self-interference.

Considering $y_{s}\left(x\right)\in {}^{M\times 1}$ is the signal vector independent and identically distributed (i.i.d) zero mean cyphers and unit variance that the $T_{x}$ will be transmitting while $y_{d}\left(x\right)\in {}^{M\times 1}$ is assumed to be the jamming noise vector of i.i.d. zero mean cyphers and unit variance the transmitter ($T_{x}$) will be transmitting. Thus, the signal vectors $R_{x}$ and Eve received can be individually described,

(8) $$z_{d}\left(x\right)=g_{sd}*\sqrt{p_{s}}*y_{s}\left(x\right)+\sqrt{\delta }g_{dd}*\sqrt{p_{d}}*y_{d}\left(x\right)+m_{d}\left(x\right),$$

(9) $$z_{e}\left(x\right)=g_{se}*\sqrt{p_{s}}*y_{s}\left(x\right)+g_{de}*\sqrt{p_{d}}*y_{d}\left(x\right)+m_{e}\left(x\right),$$

where ${}^{M\times 1}$ comprises the vector, $g_{sd},g_{se},g_{de},$ and $g_{dd}$ are explicitly described in Figure 2, and $p_{s}$ and $p_{d}$ vectors, respectively, represent the power of transmission between the $T_{x}$ and $R_{x}$. $m_{d}\left(x\right)\in {}^{M\times 1}$ and $m_{e}\left(x\right)\in {}^{M\times 1}$ are considered as the mean of independent white Gaussian noise of zero and unit variance, respectively. The multiplication and square root are used as element-wise operators, and δ represents the attenuation factor of self-interference.

Denoting the superscript (m) as a vector mth element, we formulate the respective signal-to-noise-ratios (SNRs) of the mth subcarrier at $R_{x}$ and Eve as

(10) $$\gamma_{d}^{\left(m\right)}=\frac{A_{m}y_{m}}{1+B_{m}z_{m}}\;\mathrm{and}\;\gamma_{e}^{\left(m\right)}=\frac{C_{m}y_{m}}{1+D_{m}z_{m}},$$

where $A_{m}={\left|g_{sd}^{\left(n\right)}\right|}^{2},$ $B_{m}=\delta {\left|g_{dd}^{\left(n\right)}\right|}^{2},$ $C_{m}={\left|g_{se}^{\left(n\right)}\right|}^{2},$ $D_{m}={\left|g_{de}^{\left(n\right)}\right|}^{2},$ $y_{m}=p_{s}^{\left(m\right)}$ and $z_{m}=p_{d}^{\left(m\right)}.$ Recall that as stated earlier, Eve’s CSI ($C_{m}$ and $D_{m}\forall m$) is only supposed to be recognizable by legitimate users in this Section.

Thus, the privacy capacity of this model is defined as

(11) $$S_{c}(y,z)=\frac{1}{M}\sum_{m=1}^{M}\max\left\{0,\Delta S_{m}\left(y_{m},z_{m}\right)\right\},$$

where

(12) $$\Delta S_{m}\left(y_{m},z_{m}\right)=\log\left(1+\gamma_{d}^{\left(m\right)}\right)-\log\left(1+\gamma_{e}^{\left(m\right)}\right).$$

### 4.1. Formulated Optimization Problem

In this subsection, our objective is to boost the privacy capacity of the scheme using power and rate constraints with cooperative power distribution between $T_{x}$ (source) and $R_{x}$ (destination). As compared with the conventional physical layer security techniques, the proposed SCEO and swift privacy rate optimization algorithms are suitable for the Internet of Things, because the optimization algorithms are energy efficient; therefore, the low-energy consumption necessities of IoT is enormously satisfied. Specifically, in a wireless channel transmission scheme, intrinsic noise is deployed which degrades the quality of the eavesdropper’s received signal; thus, privacy in transmission is guaranteed with no cost of supplementary power. In summary, the application of the proposed technique in IoT technologies is low power capable as it does not necessitated the use of additional energy to guarantee privacy in transmission. The authors in [38] attempted to solve this low power problem through the bisection approach by swapping the source and destination powers optimizations, iteratively. However, in this work, we provided an improved solution by jointly assigning source and destination powers, thereby formulating the first optimization problem as (13):

(13) $$\begin{array}{c} \begin{array}{c} max\\ x,y \end{array}\frac{1}{M}\sum_{m\in \Psi z}\left(\log\left(1+\frac{A_{m}y_{n}}{1+B_{m}z_{m}}\right)-\log\left(1+\frac{C_{m}y_{m}}{1+D_{m}z_{m}}\right)\right)\\ s.t.\frac{1}{M}\sum_{m\in \Psi z}\log\left(1+\frac{A_{m}y_{n}}{1+B_{m}z_{m}}\right)\geq C_{sd}\\ \sum_{m\in \Psi z}z_{m}\leq p_{d},z_{m}\geq 0\forall m\in \Psi_{z}\\ \sum_{m}y_{m}\leq p_{s},y_{m}\geq 0\forall m\in ℵ\\ z_{m}=0,\forall m\in \Psi \begin{array}{c} ⊥\\ z \end{array} \end{array}$$

where

(14) $$\begin{array}{c} \Psi_{z}=\Theta_{z}\cap \Phi \\ \Theta_{z}=\left\{m\left|\frac{A_{m}}{1+B_{m}z_{m}}>\frac{C_{m}}{1+D_{m}z_{m}},\forall m\in ℵ\right.\right\}\\ \Phi =\left\{m\left|\frac{A_{m}}{C_{m}}>\frac{B_{m}}{D_{m}},\frac{B_{m}}{D_{m}}<1,\forall m\in ℵ\right.\right\}\\ \Psi \begin{array}{c} ⊥\\ z \end{array}=\left\{m\left|m\right.\in ℵ,m\notin \Psi_{z}\right\} \end{array}$$

The privacy capacity of our system is considered as the objective function, and the approximation is performed over the privacy capacity of the group of subcarriers which warrants a positive capacity of $\psi_{z}$. First, the rate constraint which guarantees the quality of service (QoS) of the network is considered. Although the techniques warrant data exchange or channel feedback between the authentic users, which can result in a slight rate performance degradation, typically IoT devices and applications have very low data rates. Therefore, this setback of low rate performance does not alter the adoption of the scheme in IoT operations. Second, the jamming mitigation power constraint which has its summation over the set of subcarriers $\psi_{z}$ is considered. This is due to the need to properly manage power so no power waste is experienced by subcarriers which might not guarantee a positive gain of privacy. Last, the third constraint which is the power constraint at Source is taken into consideration. Furthermore, the minimum expected rate is represented as $C_{sd}$, whereas $p_{s}$ and $p_{d}$ are set to represent the optimal sum of powers at Source and Destination.

With the aim of exploring the Karush–Kuhn–Tucker (KKT) conditions, we formulated the Lagrangian function of the problem as

(15) $$\begin{array}{c} L(y,z,\lambda ,\mu_{y},\mu_{z},\upsilon_{y},\upsilon_{z})=\\ -\frac{1}{M}\sum_{m\in \psi_{z}}\left(\log\left(1+\frac{A_{m}y_{m}}{1+B_{m}z_{m}}\right)-\log\left(1+\frac{C_{m}y_{m}}{1+D_{m}z_{m}}\right)\right)\\ -\mu_{y}^{R}y-\mu_{z}^{R}z+\mu_{y}\left(\sum_{m}{y_{m}-p}_{s}\right)+\upsilon_{z}\left(\sum_{m\in \psi_{z}}{y_{m}-p}_{d}\right)\\ +\lambda \left({\tilde{C}}_{sd}-\frac{1}{M}\sum_{m\in \psi_{z}}\log(1+\frac{A_{m}y_{m}}{1+B_{m}z_{m}})\right) \end{array}$$

Considering the KKT conditions as

(16) $$\left\{\begin{array}{c} \forall m\in \psi_{z}\left\{\begin{array}{c} \frac{\partial l}{\partial y_{m}}=-\varphi_{m}\left(y_{m},z_{m}\right)-\frac{1}{M}\frac{A_{m}}{1+B_{m}z_{m}A_{m}y_{m}}-\mu_{y}\left(m\right)+\upsilon_{y}=0\\ \frac{\partial l}{\partial z_{m}}=-\varphi_{m}\left(y_{m},z_{m}\right)-\frac{1}{M}\left(\frac{B_{m}}{1+B_{m}z_{m}A_{m}y_{m}}-\frac{B_{m}}{1+B_{m}z_{m}}\right)-\mu_{z}\left(m\right)+\upsilon_{z}=0 \end{array}\right.\\ y_{m}\geq 0,\mu_{y}\left(m\right)0,y_{m}\mu_{y}\left(m\right)=0,\forall m\in ℵ\\ z_{m}\geq 0,\mu_{z}\left(m\right)0,z_{m}\mu_{z}\left(m\right)=0,\forall m\in \psi_{z}\\ \upsilon_{y}\geq 0,\sum {{}_{m}y_{m}\leq p}_{s},\upsilon_{y}\left(\sum {{}_{m}y_{m}-p}_{s}\right)=0\\ \upsilon_{z}\geq 0,\sum {{}_{\in \psi_{y}}y_{m}\leq p}_{d},\upsilon_{z}\left(\sum {{}_{\in \psi_{y}}y_{m}-p}_{d}\right)=0\\ \lambda \geq 0,{\tilde{C}}_{sd}\leq \frac{1}{M}\sum {}_{m\in }\psi_{z}\log\left(1+\frac{A_{m}y_{m}}{1+B_{m}z_{m}}\right)\\ \lambda \left({\tilde{C}}_{sd}-\frac{1}{M}\sum {}_{m\in }\psi_{z}\log\left(1+\frac{A_{m}y_{m}}{1+B_{m}z_{m}}\right)\right)=0 \end{array}\right.$$

where

(17) $$\varphi \left(y_{m},z_{m}\right)=\frac{1}{M}\frac{A_{m}}{1+B_{m}z_{m}+A_{m}y_{m}}-\frac{1}{M}\frac{C_{m}}{1+D_{m}z_{m}+C_{m}y_{m}}$$

(18) $$\vartheta_{m}\left(y_{m},z_{m}\right)=\frac{1}{M}\left(\frac{B_{m}}{1+B_{m}z_{m}+A_{m}y_{m}})-\frac{B_{m}}{1+B_{m}z_{m}}-\frac{D_{m}}{1+D_{m}z_{m}+C_{m}y_{m}}+\frac{D_{m}}{1+D_{m}z_{m}}\right)$$

Equations (16)–(18) above are the derivations of the KKT conditions which the Lagrangian function of the problem must satisfy for optimal solution to be achieved.

Apparently, attempting to find solution to the source-to-destination KKT conditions, a two-dimensional (2-D) bisection search estimation is performed on parameters λ,ν, and μ as analyzed in Algorithm 1.
**Algorithm 1** Algorithm to solve problem (15) by solving the KKT conditions of (16), using **2-D** search approach for μ, ν, and λ.**Initialize:**$A_{m},B_{m},C_{m},D_{m},y_{m},\forall m\in ℵ;p_{s};sd;C_{SOD};\varepsilon ,\zeta .$**Generate:**1:Initiate λ = 0 therefore, eliminating the rate constraint), perform search for ν and y.2:Compute C(y) as sd capacity.3:**if**$C\left(y\right)>C_{SOD}$**then**4: **revert** y (meaning the rate constraint is achieved).5:**else** **2-D** search: perform search for ν=0 to satisfy power constraint till precision ε is achieved. Perform search λ>0 for every given ν to achieve the rate constraint until precision ζ is achieved. Given each pair of λ and ν, $y_{m}\geq 0$ is achieved as the solution of (24) for every m∈ℵ.6: **revert** y (meaning the rate constraint is achieved).7:**end if**

Considering Algorithm 1 (bisection algorithm), the following set of equations which require solution will be encountered. These represent two nonlinear systems of equations:

(19) $$\left\{\begin{array}{c} \frac{\partial l}{\partial y_{m}}=-\varphi_{m}\left(y_{m},z_{m}\right)-\frac{\lambda }{M}\frac{A_{m}}{1+B_{m}z_{m}A_{m}y_{m}}-\mu_{y}\left(m\right)+\upsilon_{y}=0\\ \frac{\partial l}{\partial z_{m}}=-\varphi_{m}\left(y_{m},z_{m}\right)-\frac{\lambda }{M}\left(\frac{B_{m}}{1+B_{m}z_{m}A_{m}y_{m}}-\frac{B_{m}}{1+B_{m}z_{m}}\right)-\mu_{z}\left(m\right)+\upsilon_{z}=0 \end{array}\right..$$

Finding solution for the KKT conditions which contains these two nonlinear equations appears to be unnerving; therefore, we consider applying the approach of sequential convex approximation.

### 4.2. Sequential Convex Approximation

In attempt to realize a sequential convex approximation, the rate constraint and the objective function which constitute the optimization problem formulated in (13) is rewritten as (20) below.

(20) $$\begin{array}{c} f\left(y,z\right)=\frac{1}{M}\sum_{m\in \Theta z}\left(\log\left(1+\frac{A_{m}y_{m}}{1+B_{m}z_{m}}\right)-\log\left(1+\frac{C_{m}y_{m}}{1+D_{m}z_{m}}\right)\right)\\ =\frac{1}{M}\sum_{m\in \Theta z}\left(\log\left(1+B_{m}z_{m}+A_{m}y_{m}\right)+\log\left(1+D_{m}z_{m}\right)\right)\\ -\frac{1}{M}\sum_{m\in \Theta z}\left(\log\left(1+D_{m}z_{m}+C_{m}y_{m}\right)+\log\left(1+B_{m}z_{m}\right)\right)\\ =f_{1}\left(y,z\right)+f_{2}\left(y,z\right)\frac{1}{M}\sum_{m}\left(\log(1+B_{m}z_{m}+A_{m}y_{m})\right)\\ -\frac{1}{M}\sum_{m}\left(\log(1+B_{m}z_{m})\right)\geq C_{sd}\\ f_{3}\left(y\right)+f_{4}\left(y,z\right)\geq C_{sd} \end{array}$$

Evidently, $f_{1}$ and $f_{4}$ functions are concave while $f_{2}$ and $f_{3}$ functions are convex. Considering first-order Taylor series expansion of convex functions as the function’s underestimator. By denoting the first-order Taylor series expansion of $f_{2}$ and $f_{3}$ around $\left(y^{\left(h\right)},z^{\left(h\right)}\right)$ points and expressing them, respectively, as ${{\tilde{f}}_{2}}^{\left(h\right)},{{\tilde{f}}_{3}}^{\left(h\right)}$, the following was realized,

(21) $$\begin{array}{c} f_{1}(y,z)+f_{2}(y,z)\geq f_{1}(y,z)+{{\tilde{f}}_{2}}^{\left(h\right)}(y,z)\forall y,z\in {}^{M}\\ f_{4}(y,z)+f_{3}(z)\geq f_{4}(y,z)+{{\tilde{f}}_{3}}^{\left(h\right)}(z)\forall y,z\in {}^{M} \end{array}$$

where

(22) $$\begin{array}{c} {{\tilde{f}}_{2}}^{\left(h\right)}(y,z)=f_{2}(y^{\left(h\right)},z^{\left(h\right)})+\nabla f_{2}(y^{\left(h\right)},z^{\left(h\right)}{)}^{R}(\left[\begin{array}{c} y\\ z \end{array}\right]\\ -\left[\begin{array}{c} y^{\left(h\right)}\\ z^{\left(h\right)} \end{array}\right])\\ {{\tilde{f}}_{3}}^{\left(h\right)}(z)=f_{3}(z^{\left(h\right)})+\nabla f_{3}(z^{\left(h\right)}{)}^{R}(z-z^{\left(h\right)})\\ \nabla f_{2}(y,z)=-\frac{1}{M}\left[\begin{array}{c} \begin{array}{c} \frac{C_{1}}{1+D_{1}z_{1}+C_{1}y_{1}}\\ \vdots \\ \frac{C_{M}}{1+D_{M}z_{M}+C_{M}y_{M}} \end{array}\\ \begin{array}{c} \frac{D_{1}}{1+D_{1}z_{1}+C_{1}y_{1}}+\frac{B_{1}}{1+B_{1}z_{1}}\\ \vdots \\ \frac{D_{M}}{1+D_{M}z_{M}+C_{M}y_{M}}+\frac{B_{M}}{1+B_{M}z_{M}} \end{array} \end{array}\right]\\ \nabla f_{3}(z)=-\frac{1}{M}\left[\begin{array}{c} \frac{B_{1}}{1+B_{1}z_{1}}\\ \vdots \\ \frac{B_{M}}{1+B_{M}z_{M}} \end{array}\right] \end{array}$$

Based on work done in [34] and assuming $\frac{B_{m}}{D_{m}}<1$ we further simplify $\Theta_{z}$ and obtain

(23) $$\begin{array}{c} \Theta_{z}=\left\{m\left|z_{m}\geq 0,\frac{A_{m}}{1+B_{m}z_{m}}>\frac{C_{m}}{1+D_{m}z_{m}},\forall m\in ℵ\right.\right\}\\ =\left\{m\left|\left(B_{m}C_{m}-A_{m}D_{m}\right)z_{m}>A_{m}-C_{m},\forall m\in ℵ\right.\right\}\\ =\left\{m\left|z_{m}\geq \frac{A_{m}-C_{m}}{B_{m}C_{m}-A_{m}D_{m}},1>\frac{A_{m}}{C_{m}}>\frac{B_{m}}{D_{m}},\forall m\in ℵ\right.\right\}\\ \cup \left\{m\left|z_{m}\geq 0,1<\frac{A_{m}}{C_{m}}>\frac{B_{m}}{D_{m}},\forall m\in ℵ\right.\right\} \end{array}$$

Thus, following iteration *h*, we formulate the optimization problem as follows,

(24) $$\begin{array}{c} \begin{array}{c} \max\\ y,z \end{array}f_{1}\left(y,z\right)+{{\tilde{f}}_{2}}^{\left(h\right)}\left(y,z\right)\\ s.t.f_{4}\left(y,z\right)+{{\tilde{f}}_{3}}^{\left(h\right)}(z)\geq C_{sd}\\ \sum_{m}z_{m}\leq p_{d},z_{m}\geq 0\forall m\in ℵ\\ z_{m}=0\forall m\in \left\{m\left|\frac{A{}_{m}}{C_{m}}<\frac{B_{m}}{D_{m}}<1,\forall m\in ℵ\right.\right\}\\ \cup \left\{m\left|\frac{B_{m}}{D_{m}}>1,\forall m\in ℵ\right.\right\}\\ z_{m}\geq \frac{A{}_{m}-C_{m}}{B_{m}C_{m}-A_{m}D_{m}}\forall m\in \left\{m\left|1>\frac{A_{m}}{C_{m}}>\frac{B_{m}}{D_{m}},\forall m\in ℵ\right.\right\}\\ \sum_{m}y_{m}\leq p_{s},y_{m}\geq 0\forall m\in ℵ \end{array}$$

It is observed that a convex optimization problem occurs at each point of iteration. This attempts to optimize a lower bound on the primal objective function and guarantees the rate constraint. As the iterative Algorithm reaches convergence, a decent approximation on the optimal values is expected. The optimization process is detailed in Algorithm 2.
**Algorithm 2** Sequential Convex Estimation Optimization Algorithm for solving optimization problem.**Initialize:**$\left(A_{m},B_{m},C_{m},D_{m}\right):\forall_{m}\in ℵ,p_{d},p_{s},C_{sd},\zeta .$**Generate:**$y^{\left(0\right)}=\frac{1}{M}p_{s}1_{M}$$z^{\left(0\right)}=\frac{1}{\left|\Theta_{x}\right|}p_{d}a\left(i\right)=1$1:**if**$\left(i\in \Theta_{x}\right)$2:**else**a(i)=0,t=13:**While***True***do**4:**end if** Perform the convex optimization problem in (18) to realize $y^{\left(t\right)}$, $z^{\left(t\right)}$, **if** $\left(∥[y^{\left(t\right)};z^{\left(t\right)}]-\left[y^{\left(t-1\right)};z^{\left(t-1\right)}\right]∥<\zeta \right)$ **then**
  break. **else**  t=t+1
 **end if**5:**end**6:return $y^{\left(t\right)}$, $z^{\left(t\right)}$;

### 4.3. Optimization of Swift Privacy Rate in a MIMOME

Considering a network scenario where multiple eavesdroppers operate, the first assumption in realizing a swift privacy rate will be that Eve has full knowledge of her covariance matrix of noise and interference $(M_{E})$ just as she has full knowledge of all her entire CSIs (as earlier stated); then, she can make use of the optimal receiving antenna and her achievable rate would be derived as

(25) $$R_{TE}=\log\left|M_{E}+aA_{1}Z_{r}A_{1}^{G}\right|-\log\left|M_{E}\right|$$

where

(26) $$M_{E}=I+\frac{aPm}{A_{Tx}-r}A_{2}A_{2}^{G}+\frac{eP_{B}}{A_{Rx}}BB^{G}$$

Then, the swift realizable privacy rate would be expressed as

(27) $$P_{R}={\left(R_{TR}-R_{TE}\right)}^{+}$$

However, for a multiple-input multiple-output multiple-antenna eavesdropping (MIMOME) system, assuming there is a constraint on the number of Eve’s antenna $\left(A_{E}\right)$, one major issue of the system will be on how can to optimize the parameters of the transmission in such a way that Eve’s interference in the transmission is made difficult. This becomes our focus in this subsection.

In this MIMOME system, if there is great increase in of $\left(A_{E}\right)$, the artificial noise does not have a significant impact on the rate of transmission between $T_{x}$ and Eve $\left(R_{TE}\right)$. Therefore, the assumption in this subsection is that there is a constraint on Eve’s maximum number of antennas; therefore, for the reason of optimization, a worst case is assumed, and thus this maximum number of antennas is considered as $\left(A_{E}\right)$.

To achieve this optimization, $T_{x}$ and $R_{x}$ cannot utilize the swift CSI at Eve, so as earlier mentioned, $P_{R}$ is inappropriate for this optimization. From some previous research [6,13,18], it is observed that Eve’s symptotic rate is a decent approximation to the tangible rate; thus, we adopted this fact in order to achieve optimization. Suppose that the we make no assumption on the realizable random parameter (r) and assuming that $z_{r}$ is a vector encompassing the transverse elements of $Z_{r}$, and $\left(A_{E}\right)$ is discreetly huge. Then, by rewriting (26), we obtained

(28) $$\begin{array}{c} R_{TE}=\log\left|I+\frac{aPm}{A_{Tx}-r}A_{2}A_{2}^{G}+\frac{eP_{B}}{A_{Rx}}BB^{G}+aA_{1}z_{r}A_{1}^{G}\right|\\ -\log\left|I+\frac{aPm}{A_{Tx}-r}A_{2}A_{2}^{G}+\frac{eP_{B}}{A_{Rx}}BB^{G}\right|\\ =\log\left|I+K_{3}{\bar{\Theta }}_{3}K_{3}^{G}\right|-\log\left|I+K_{4}{\bar{\Theta }}_{4}K_{4}^{G}\right|, \end{array}$$

as $K_{3}$ and $K_{4}$ are expanded as $K_{3}=\frac{1}{\sqrt{A_{E}}}\left[A_{1},A_{2}A_{3}\right],$ and $K_{4}=\frac{1}{\sqrt{A_{E}}}\left[A_{2},B\right].$ where

(29) $${\bar{\Theta }}_{3}=A_{E}\mathrm{trans}\left(\left[az_{r}^{P},\frac{aP_{m}}{A_{Tx}-r}1A_{Tx}-r,\frac{bP_{B}}{A_{Rx}}1A_{Rx}\right]\right),$$

(30) $${\bar{\Theta }}_{4}=A_{E}\mathrm{trans}\left(\left[\frac{aP_{m}}{A_{Tx}-r}1A_{Tx}-r,\frac{bP_{B}}{A_{Rx}}1A_{Rx}\right]\right),$$

Deriving from the approximation of $R_{TE}$, an objective function which entails the approximation of $P_{R}$ is proposed. Note that the proposed objective function does not depend on the CSI of Eve; however, it considers the approximate swift rate of Eve. Likewise, as it is assumed that the transmitter and receiver has full knowledge of the null space (G), the receivers exact rate is applied but not in its asymptotic form. The objective function is expressed as follows,

(31) $$\begin{array}{c} h\left(z_{r},P_{m},P_{B}\right)=-\log\left|M_{B}+GV_{1}Z_{r}V_{1}^{G}G^{G}\right|+\log\left|M_{B}\right|\\ +A_{E}\Omega \left({\bar{\beta }}_{3},{\bar{\Theta }}_{3},{\bar{\sigma }}_{3}\right)-A_{E}\Omega \left({\bar{\beta }}_{4},{\bar{\Theta }}_{4},{\bar{\sigma }}_{4}\right), \end{array}$$

where $M_{B}$ implies that the receiver has full knowledge of her covariance matrix of noise and interference, ${\bar{\beta }}_{3}=\frac{A_{Tx}+A_{Rx}}{A_{E}}$, ${\bar{\beta }}_{4}=\frac{A_{Tx}-r+A_{Rx}}{A_{E}}$, while ${\bar{\sigma }}_{3}$ and ${\bar{\sigma }}_{4}$ are solutions to the formulated problem. In order to optimize the swift privacy rate, we proposed an optimization problem as stated below.

(32) $$\begin{array}{c} \overset{\min}{r}\overset{\min}{z_{r},P_{m},P_{B}}h\left(z_{r},P_{m},P_{B}\right)\\ s.t.\sum_{i=1}^{r}z_{r}\left(i\right)+P_{m}\leq P_{A}^{\max}\\ z_{r}\left(i\right)\geq 0,\forall i=1,...,r\\ P_{m}\geq 0\\ 0\leq P_{B}\leq P_{B}^{\max} \end{array}$$

In the optimization problem above, the constraints are in their convex form; nevertheless, h(.) is yet to assume a convex form. We can achieve this by rewriting the function

(33) $$\begin{array}{c} h\left(z_{r},P_{m},P\right)=-\log\left|M_{B}+GV_{1}Z_{r}V_{1}^{G}G^{G}\right|+\log\left|M_{B}\right|\\ +A_{E}{\bar{\beta }}_{3}\upsilon_{\Theta_{3}}\left({\bar{\sigma }}_{3}\right)-A_{E}{\bar{\beta }}_{4}\upsilon_{\Theta_{4}}\left({\bar{\sigma }}_{4}\right)\\ +A_{E}\log\left(\frac{{\bar{\sigma }}_{4}}{{\bar{\sigma }}_{3}}\right)+A_{E}\left({\bar{\sigma }}_{3}-{\bar{\sigma }}_{4}\right)\log\left(c\right)\\ =-\log\left|M_{B}+GV_{1}Z_{r}V_{1}^{G}G^{G}\right|+\log\left|M_{B}\right|\\ +\sum_{k=1}^{N_{{\bar{\Theta }}_{3}}}\log\left(1+{\bar{\sigma }}_{3}\left({\bar{\Theta }}_{3}\right)k,k\right)-\sum_{k=1}^{N_{{\bar{\Theta }}_{4}}}\log\left(1+{\bar{\sigma }}_{4}\left({\bar{\Theta }}_{4}\right)k,k\right)\\ +A_{E}\log\left(\frac{{\bar{\sigma }}_{4}}{{\bar{\sigma }}_{3}}\right)+A_{E}\left({\bar{\sigma }}_{3}-{\bar{\sigma }}_{4}\right)\log\left(c\right) \end{array}$$

From Equation (33), we can linearize $+\sum {}_{k=1}^{N_{{\bar{\Theta }}_{3}}}\log\left(1+{\bar{\sigma }}_{3}\left({\bar{\Theta }}_{3}\right)k,k\right)$, which is the convex form of h(.) at every iteration point of the optimization algorithm using the expansion method of first-order Taylor series. Similarly, we can resolve the reliance state of h(.) on the ${\bar{\sigma }}_{3},{\bar{\sigma }}_{4}$ parameters by making them constant with respect to their values and the upgrading them at the end to achieve the following,

(34) $$1-{\bar{\sigma }}_{i}^{j+1}=\frac{{\bar{\beta }}_{i}{\bar{\sigma }}_{i}^{j+1}}{N_{{\bar{\Theta }}_{i}}}\sum_{k=1}^{N_{{\bar{\Theta }}_{i}}}\frac{({\bar{\Theta }}_{i}^{j+1})k,k}{1+{\bar{\sigma }}_{i}^{j+1}\left({\bar{\Theta }}_{i}^{j+1}\right)k,k},i=3,4.$$

note that j+1 represents the parameter value at j+1 iteration. Thus, we optimized the preceding convex function at *j* iteration,

(35) $$\begin{array}{c} g^{j}\left(y_{r}\right)=-\log\left|M_{B}+GV_{1}Z_{r}V_{1}^{G}G^{G}\right|\\ -\sum_{k=1}^{N_{{\bar{\Theta }}_{4}}}\log\left(1+{\bar{\sigma }}_{{}_{3}}^{j}\left({\bar{\Theta }}_{4}\right)k,k\right)+{\left(y_{r}-y_{{}_{r}}^{j}\right)}^{J}\nabla_{y_{r}}f^{j}\left|{}_{y_{r}=y_{{}_{r}}^{j}}\right., \end{array}$$

denoting $y_{r}={\left[z_{r}^{J},P_{m},P_{B}\right]}^{J}$ and $f^{j}\left(y_{r}\right)=\log\left|M_{B}\right|+\sum_{k=1}^{N_{{\bar{\Theta }}_{3}}}\log\left(1+{\bar{\sigma }}_{{}_{3}}^{j}\left({\bar{\Theta }}_{3}\right)k,k\right)$. Recall that terms that are constant were not used in (25) because they do not have any effect in the optimization. However, at this point, h(.) has assumed a convex function and we can then optimize it using the preceding optimization

(36) $$\begin{array}{c} y_{r}^{j+1}=\overset{\arg\min}{y_{r}}g^{j}\left(y_{r}\right)\\ s.t.\sum_{i=1}^{r}z_{r}\left(i\right)+P_{m}\leq P_{A}^{\max}\\ z_{r}\left(i\right)\geq 0,\forall i=1,...,r\\ P_{m}\geq 0\\ 0\leq P_{B}\leq P_{B}^{\max}. \end{array}$$

Similarly, at this point, if any possible values of *r* is deployed, an optimal output will be recorded. The summary of our proposed optimization procedure can be seen in Algorithm 3. Although different optimization methods to tackle this kind of problem were proposed in [13,18] comparatively, these methods give almost similar outcome, nonetheless, our proposed Algorithm 3 significantly executes with minimal complexity. Our figures illustrate the efficiency and out-performance of our swift optimization algorithm as compared with nonoptimal parameters.
**Algorithm 3** Swift privacy rate optimization algorithm.**Input actual**:ε, ${\bar{\sigma }}_{3}^{0}$, ${\bar{\sigma }}_{4}^{0}$, and initiate $h^{\min}=0$1:**for**$r=1:A_{Tx}$**do**2: Set $y_{r}$ to satisfy the constraints3: **while** $\frac{∥y_{r}^{j}-y_{r}^{j-1}∥}{∥y_{r}^{j-1}∥}>\varepsilon$ **do**
4:  execute (28) till $y_{r}^{j+1}$ is realized5:  execute (25) using $y_{r}^{j-1}$ till ${\bar{\sigma }}_{3}^{0}$, ${\bar{\sigma }}_{4}^{0}$ is updated6:  j=j+1.
7: **end while**
8: **if** $h\left(y_{r}^{j}\right)<h^{\min}$ **then**
9:  $h^{\min}=h\left(y_{r}^{j}\right)$
10:   $y^{\min}=y_{r}^{j}$
11: **end if**
12:**end for**13:Revert $y^{\min}$.

## 5. Numerical Analysis and Results

In this section, MATLAB simulation results based on our proposed SCEO algorithm and the Swift privacy rate optimization algorithm is presented. Our investigations show that the magnitudes of all transmission channels are distributed in a Rayleigh form with an even unit of mean square, while the attenuation factor of the transmission self-interference ρ is set to be 0.8, except where slight changes are required. The transmission power constraints were set at 20db except where otherwise stated.

### 5.1. Realized Privacy Capacity

**Figure 3:**
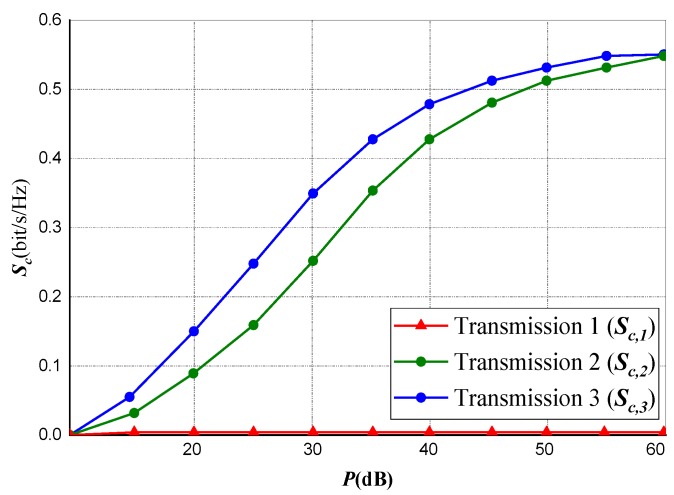
Realized privacy capacity against eavesdroppers under power and rate constraints.

**Figure 4:**
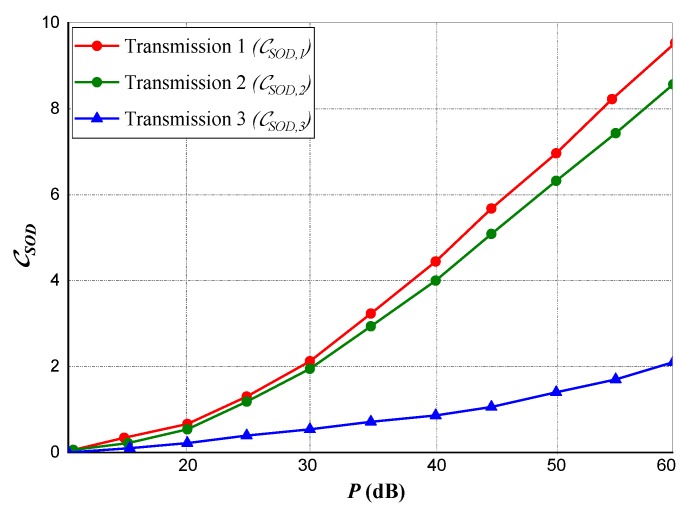
Realized privacy capacity for Source-to-Destination $\left(C_{SOD}^{*}=0.8C_{SOD,1}\right)$ transmission under power and rate constraints.

We performed a transmission performance evaluation for a three-node transmission under power and rate constraints as shown in Figure 3 and Figure 4. A comparison of three separate transmission scenarios with respect to the privacy capacity of their transmissions is shown in the experiment.

Figure 3 indicates privacy capacity against Eve, whereas Figure 4 represents the data rate of the transmission between the source and destination *(Source-to-Destination)* specifically realizing for the entire $A_{m}<C_{m},\forall m\in ℵ$. This clearly implies that, for the entire set of subcarriers, the Eve’s channel is stronger from the Source $\left(T_{x}\right)$ than the Destination $\left(R_{x}\right)$ has from the Source.

For Transmission 1 (the first transmission scenario), at the Source and Destination terminals, data rate is optimized subject to power constraints. However, this occurs without the constraint for privacy capacity. The subsequent obtainable data rate is signified by $C_{SOD,1}$. On the other hand, as expected for this channel, the subsequent privacy capacity $S_{c,1}$ is realized as zero.

For Transmission 2, the transmissions privacy capacity is optimized subject to power constraints at *Source* and *Destination* likewise a rate constraint of *Source-to-Destination*. Setting the lesser bound on the rate (i.e., the constrained rate) at $C_{SOD}^{*}=0.8C_{SOD,1}$, the equivalent rate realized at the channel transmission is signified by $C_{SOD,2}$, as anticipated, the curve is vague from that of $C_{SOD}^{*}$. $S_{c,2}$ represents the subsequent attained transmission privacy capacity and it is huge and quite close to Transmission 3.

In Transmission 3 (the third transmission scenario), the privacy capacity is optimized with only power constraint at *Source* and *Destination* without considering any rate constraint. Subsequently, we represented the privacy capacity as $S_{c,3}$ while the data transmission rate is signified as $C_{SOD,3}$.

Considering the three transmission scenarios, it is observed that the result obtained at the second scenario *(Transmission 2)* outperform the other two transmission scenarios in terms of *source-to-destination* data rate trade-off and the transmission’s privacy capacity.

### 5.2. Joint Power Assignment for Multiple Destinations

**Figure 5:**
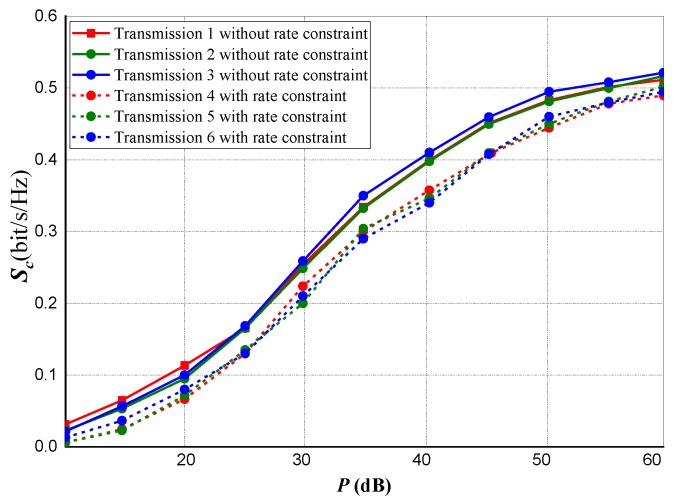
Maximum realized privacy capacity for multiple destinations transmission.

**Figure 6:**
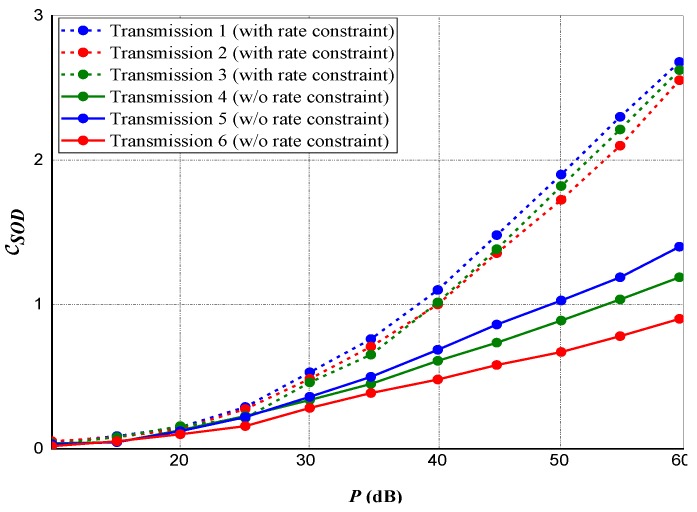
*Source-to-Destination* data rate for multiple destinations transmission.

For a multiple transmission destinations scenario, we considered $C_{n}=\chi C_{n}^{*}$, representing $C_{n}^{*}$ as the optimal data rate attainable from *Source* to *Destination* when power $\frac{p_{s}}{N}$ is assigned to *Source* for data broadcast to the nth destination. Considering χ=0.8 and N=4, Figure 5 and Figure 6, respectively, represent the maximum attained privacy capacities and data rates from the *source-to-destination*. Likewise, the equivalent outputs with no rate constraint are represented in the two figures. It is observed that if rate constraints are applied, a small measure of privacy capacities is lost, however, significant data rates are gained and maintained. For both Figure 5 and Figure 6, under rate constraint, both the achieved and constrained rates are separable.

### 5.3. Joint Power Assignment for Multiple Sources

**Figure 7:**
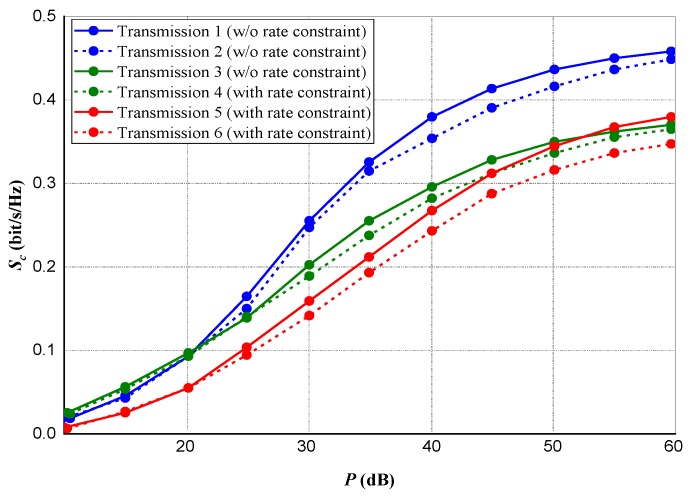
Maximum realized privacy capacity for multiple sources transmission.

**Figure 8:**
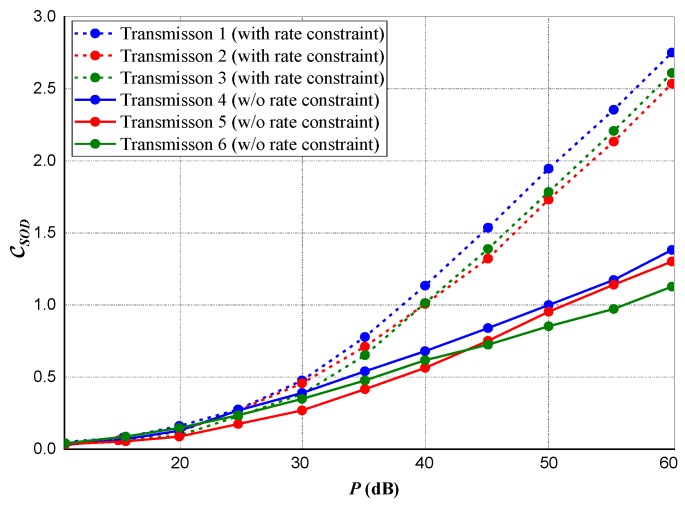
*Source-to-Destination* data rate for multiple sources transmission.

For a multiple transmission source scenario, considering the nth transmission source, we set the rate constraint as $C_{n}=\chi C_{n}^{*}$, representing $C_{n}^{*}$ as the optimal data rate attainable from *Source* to *Destination* when power $p_{s,n}=\frac{p_{s}}{M}$ is assigned to *Destination* for data broadcast from the nth *Source*. Figure 7 and Figure 8 present the data rates and privacy capacities for all three different sources. The subsequent result shows that although a small measure of privacy capacity is lost in the absence of rate constraint, significant data rates are gained and maintained.

### 5.4. Performance Comparison of Difference Algorithms

**Figure 9:**
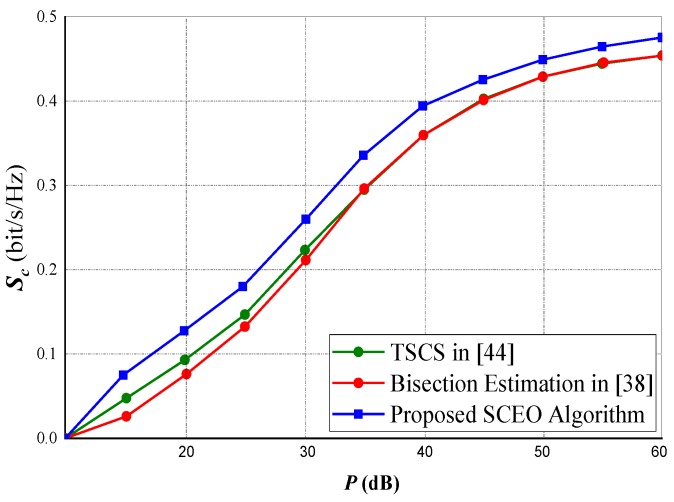
Performance of the algorithms under different power constraints.

**Figure 10:**
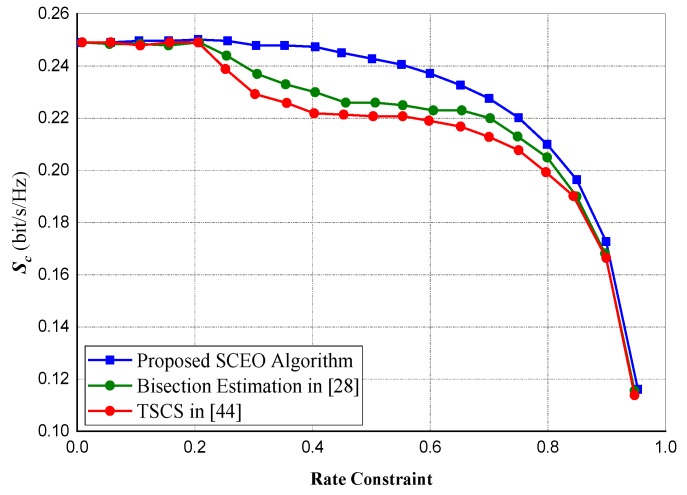
Performance of the algorithms with different rate constraints for p=20 dB.

In Figure 9 and Figure 10, we compared our proposed sequential convex estimation optimization (SCEO), which is intended to mitigate the optimization problem in (15) against the Bisection method in [38] and the two-stage cooperative jamming scheme (TSCS) in [44]. A total of 60 transmitting antennas were selected for both experiments. For the rate constraints, about 0.8 of optimal attainable capacity (OAA) was selected for Figure 9, whereas for Figure 10, the power constraint $\left(p_{s}=p_{d}=p\right)$ is set at 20 dB.

Figure 9 indicates that mostly if the power constraints are low, our proposed *SCEO* technique is latent enough to obtain optimal values. Comparing the three different techniques in Figure 10 at different variations of rate constraint. Our technique is observed to outperform the other two compared techniques notwithstanding severe rate constraints. Moreover, as the rate constraints becomes more severe, convergence might be difficult for the TSCS and Bisection techniques but our algorithm converges almost seamlessly.

**Figure 11:**
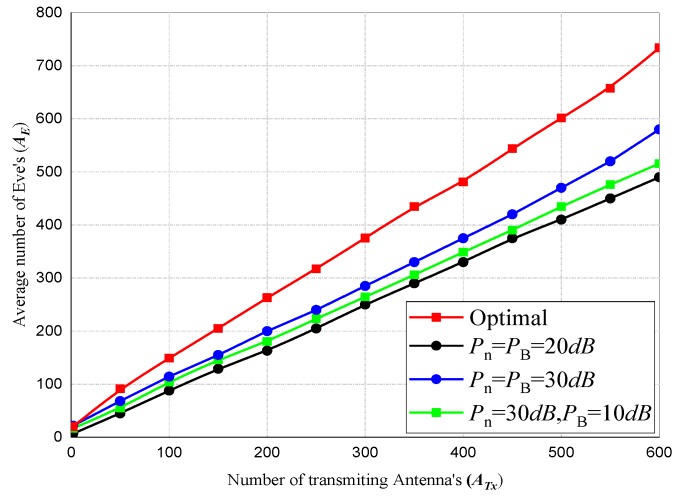
Comparison of ${\bar{A}}_{E}$ versus ${\bar{A}}_{Tx}$ with different parameters.

Finally, the complexity analysis of our swift privacy rate optimization algorithm in a multiple transmission and multiple eavesdropper scenario is shown in Figure 11. We set the optimization parameters as $A_{E}=A_{Tx},\beta_{1}=6,P_{n}^{\max}=P_{B}^{\max}=20$ dB. From the result of our investigation, there were no evident patterns in the optimal values, in addition, the optimal parameters are dependent on the outputs of G. Nevertheless, $P_{n}$ can be assumed to be frequently and approximately distributed evenly among the channels. Finally, it is observed that as $A_{E}$ becomes larger, the minimal $P_{B}$ and *r* are respectively favored by the optimization.

## 6. Conclusions

In this study, we explore the privacy capacity of wireless transmitting networks in several schemes relating to full-duplex jamming. Subject to both the transmission rate and power constraints, we considered and implemented an efficient power allocation optimization algorithm for enhancing privacy capacity in a three-node transmitting network and also in a real life MIMOME scenario. The applications of the resulting research and results can be applied to current wireless communication networks seen in rampant use in both IoT and 5G networks. Our experimental results showed that by using the sequential convex estimation optimization (SCEO) algorithm, a more optimal result and enhanced convergence is achieved. However, due to possible challenges envisaged when a multiple eavesdropper is active in a network, we expanded our research to develop a swift privacy rate optimization algorithm, which executes significantly with minimal complexity when compared with nonoptimal parameters. The use of the rate constraint together with self-interference of the full-duplex at the receiving node makes the performance of our technique outstanding from the recent studies reviewed. Furthermore, we extended our study to consider a scenario where multiple sources and multiple destinations are in use. Finally, our technique indicates that as the iterative algorithm reaches convergence, a decent approximation on the optimal values is achieved. In a future work, we intend to consider a stochastic optimization approach for privacy capacity with Eve’s CSI Unknown to Users.
